# Concentrations of Lead, Mercury, Selenium, and Manganese in Blood and Hand Grip Strength among Adults Living in the United States (NHANES 2011–2014)

**DOI:** 10.3390/toxics9080189

**Published:** 2021-08-17

**Authors:** M. Corinaud J. Gbemavo, Maryse F. Bouchard

**Affiliations:** 1 Department Environmental and Occupational Health, School of Public Health, Université de Montréal, Montreal, QC H3C 3J7, Canada; mahude.corinaud.josue.gbemavo@umontreal.ca; 2 CHU Sainte-Justine Hospital Research Center, Montreal, QC H3T 1C5, Canada

**Keywords:** grip strength, metals, neuromotor system, neurotoxicity, NHANES

## Abstract

Exposure to lead and mercury can cause deficits in neuromotor function. Selenium and manganese are essential elements, hence both deficiency and excess could result in decreased neuromotor function. We aimed to examine hand grip strength, a marker of neuromotor function, and blood concentrations of lead, mercury, selenium, and manganese in the general U.S. population. We used data from the National Health and Nutrition Examination Survey (NHANES, 2011–2014) on 6199 participants ages 20–79 years. We assessed associations of blood concentration for these elements and grip strength with generalized regression models, and cubic splines to detect possible nonlinear relations, adjusting for confounders. The results showed that mercury and manganese were not associated with grip strength. Lead was associated with weaker grip strength in women (for 10-fold increase in lead, −2.4 kg; 95% CI: −4.2, −0.5), but not in men. Higher selenium was associated with stronger grip strength in women (8.5 kg; 95% CI: 1.9, 15.1) and men (4.6; 95% CI: −11.9, 21.0), although the association was not significant in the latter. In conclusion, lead exposure was associated with weaker grip strength in women, even at the low exposure levels in the population. Furthermore, low blood selenium level was associated with weaker grip strength, suggesting that some individuals might have selenium deficiency manifesting with poorer neuromotor function.

## 1. Introduction

Environmental exposure to some metals may lead to neurotoxic effects that can manifest by alterations in different functions of the nervous system, such as cognitive impairments, mental health or mood disorders, and impairment in neuromotor function. For instance, the adverse effects of lead exposure on the nervous system have been very well documented, especially during development [1] and in adults with high levels of exposure in the workplace [2,3]. Fewer studies have addressed potential adverse effects on neuromotor function at the lower exposure levels found in adults from the general population. Blood lead concentration was associated with weaker hand grip strength in a community-based study among men in unadjusted models, but not after adjustment for covariates [4]. However, higher blood lead was also associated with other neuromotor function indicators, such as poor fine motor skills, assessed with a handwriting test, and with slower walking speed in older individuals from the general population [5,6].

Mercury is a global pollutant with known neurotoxic properties. The greatest risks for the integrity of the nervous system are thought to arise from exposure to methylmercury, especially when exposure occurs during the perinatal period [7]. Some studies also reported that high exposure during adulthood, such as in residents of Minamata, could cause severe neuromotor deficits such as cerebellar ataxia and poor motor strength [8]. Another study reported poorer motor strength and motor dexterity with higher hair methylmercury levels in adults consuming mercury-contaminated fish from the Amazonian basin [9]. However, much fewer data are available on the potential effects on neuromotor function arising from low-level exposure during adulthood.

Selenium and manganese are essential nutrients, but intake of these elements at levels exceeding the homeostatic capacity may cause toxic effects. Both elements are constituents of enzymes protecting against oxidative damage in cells [10,11], and thus could be important for protecting brain and muscle cells implicated in normal neuromotor function. Selenium is quite abundant in a normal diet, being found in a diversity of foods, including meat, seafood, and grains [11]. A few studies in elderly populations reported that lower levels of blood selenium were associated with poorer coordination, slower motor speed [12], and weaker grip strength [13]. Elevated exposure to selenium can also exert toxic effects, and it has been estimated that a narrow range of intake separates deficiency from toxicity [14]. Selenium acute toxicity was reported in individuals who consumed misformulated supplement products [14], including symptoms of fatigue and muscle pain lasting several years after the consumption of the faulty supplements. However, high intake of selenium has not been linked to overt toxicity in populations living in areas with selenium-rich soils in the United States [15] and China [16].

An adequate intake of manganese is also necessary for normal function of the nervous system, with both deficiency and excess possibly related to adverse nervous system effects [10]. The main source of exposure to the general population is through the diet, with this element being present in many foods. The neurotoxicity of this metal has been well-described in workers exposed to airborne manganese particles, which can induce a neurodegenerative syndrome similar to Parkinson’s disease [17]. Evidence of a neurotoxic effect in adults exposed to environmental sources (non-occupational) is sparse, but could occur in some populations, such as those living in the vicinity of metallurgical industries [18] and mines [19], and in those consuming manganese-contaminated drinking water [20]. However, no study has yet investigated potential nervous system deficits in relation to exposure to manganese in the general adult population with low, background exposure levels.

In summary, there are few reports on potential neurotoxicity associated with exposure to lead, mercury, selenium, and manganese in adults from the general population, as most studies focused on children or special populations with elevated exposure levels. Hence, the aim of our study was to examine the association between hand grip strength, a marker of neuromotor function, and blood concentrations of lead, mercury, selenium, and manganese in the general U.S. population. We relied on data from the U.S. National Health and Nutrition Examination Survey (NHANES), which provided measures of hand grip strength. Motor weakness is one of the most common effects of exposure to several neurotoxicants. Hence, the grip strength test is often used in the assessment of neurotoxicity in humans as well as in animal models [21].

We hypothesized that higher blood levels of lead and mercury might be associated with weaker hand grip strength. As selenium and manganese are essential nutrients at low levels, but can also have neurotoxic effects at higher levels, we hypothesized that the relation between blood concentrations and grip strength might display an inverse U-shaped curve.

## 2. Materials and Methods

### 2.1. Study Population

We used data collected during the cycles 2011–2012 and 2013–2014 of the NHANES, a nationally representative of the non-institutionalized U.S. population. Data were gathered from respondents through questionnaires, a medical examination in mobile examination, and laboratory analysis of biological samples. Among the 19,931 respondents of NHANES cycles 2011–2012 and 2013–2014, 6199 met the following inclusion criteria: (i) age between 20 and 79 years; (ii) data on grip strength of both hands; and (iii) data on blood concentrations of lead, mercury, manganese, and selenium. We excluded participants without valid hand grip measurements (*n* = 180 with incorrect arm/hand position during the test) and those who reported having had a hand surgery (*n* = 203). The study was approved by the National Center for Health Statistics Research Ethics Review Board and participants gave signed informed consent [22].

### 2.2. Measurement of Blood Metals

A sample of venous blood was collected from each survey participant during the physical examination; frozen at −20 °C; and shipped to the Division of Laboratory Sciences, National Center for Environmental Health, CDC (Atlanta, GA, USA) for analysis. The concentrations of lead, mercury, selenium, and manganese were measured using an inductively coupled plasma-mass spectrometer dynamic reaction cell (Elan ICP-DRC-MS instrument). State-of-the-art quality controls were applied, including standard reference materials for external calibration and spiked pools for internal quality control. NHANES reports blood concentrations for inorganic mercury and total mercury (the difference representing organic mercury). Although organic and inorganic forms of mercury can have a differential impact on the nervous system, both have neurotoxic properties. Hence, we only used total mercury in our analysis. Further details on the laboratory procedures are available elsewhere [23].

### 2.3. Measurement of Hand Grip Strength

Grip strength was evaluated using the Hand Dynamometer (Takei Digital gripper force gauge model T.K.K.5401) to obtain a measurement in kg of the maximum force exerted by hands. Before measurement of handgrip strength, the dynamometer was adjusted to participants’ hand size. Participants were instructed to squeeze the dynamometer as hard as possible using one hand. The test was repeated three times for each hand, alternating hands with a 60 s rest between measurements on the same hand. The measures were considered valid when the participant was able to perform the test while standing and managed to form a 90° angle with the index on the dynamometer handle. In our analysis, we used the combined hands’ grip strength, representing the sum of the largest reading from each hand. Further details on the grip strength test procedures are available elsewhere [24].

### 2.4. Potential Confounders

The potential confounding factors considered were as follows: age (in years), education (less than 9th grade; 9–12th grade (no diploma); high school graduate/GED equivalent; some college or associate degree; college graduate or above), race/ethnicity (non-Hispanic White; non-Hispanic Black; non-Hispanic Asian and other non-Hispanic groups; Mexican American and other Hispanics), family income to poverty ratio grouped into quartiles (<0.87, 0.87–1.65, 1.65–3.58, >3.58), body mass index (BMI, in kg/m^2^), smoking (never, occasional, regular smoker), and alcohol consumption (no, yes). Participants who smoked less than 100 cigarettes in their lifetime were labelled as ‘never smokers’; those who smoked cigarettes, pipes, cigars, little cigars, or electronic cigarettes during the past 5 days were ‘regular smokers’; those who did not smoke in the last 5 days were ‘occasional smokers’. For alcohol consumption, participants were grouped into ‘yes’ if they reported consuming >12 alcoholic beverages in the past year, and into ‘no’ otherwise.

### 2.5. Statistical Analyses

All analysis were stratified by sex because there is a large difference in hand grip strength between men and women and because previous studies have reported differential susceptibility to metal neurotoxicity in men and women (e.g., [25]). Given the large sample size, these sex-stratified analyses could be performed with sufficient statistical power. For descriptive purposes, we analyzed whether participant characteristics were associated with grip strength, using ANOVA or Kruskal Wallis test (for between-groups unequal and equal variance, respectively).

The main outcome was the combined hands’ grip strength, representing the sum of the largest dynamometer reading from each hand. We used generalized regression models (GLMs) for complex survey samples to analyze the association between blood concentrations of elements (lead, mercury, selenium, and manganese) and grip strength, adjusting for confounding factors. Each element concentration in blood was modelled separately and a sensitivity analysis was carried out by including all four elements in the models to produce association estimates for mutually-adjusted elements. Blood concentrations of all elements were log10-transformed to normalize the distribution and entered as continuous values in models. Age, education, BMI, and race/ethnicity were including a priori in all models because they are strong predictors of grip strength [26]. Additional covariates were included in models when associated in univariate analysis with both grip strength and at least one blood concentration of the following elements (at *p* < 0.20): family income-to-poverty ratio, smoking status, and alcohol consumption. All models were adjusted for the same covariates. We also ran restricted cubic splines models to generate plots showing the shape of the associations, setting 5 knots, placed at the 5th, 25th, 50th, 75th, and 95th percentiles. This allows for a visual exploration of the data to detect thresholds in the associations, or inverse ‘U-shaped’ curve that may characterize associations for essential nutrients (i.e., manganese and selenium).

In addition to the GLMs on the outcome for combined hands’ grip strength (analyzed as continuous values), we also ran analyses on grip strength categorized into more clinically relevant groups. In order to do this, we standardized grip strength measures for age and sex by calculating z-scores, and then we created two groups: ‘low’ and ‘normal’ grip strength, defined as a z-score ≤20th percentile and >20th percentile, respectively. This cut-off to define motor weakness based on hand dynamometer measures of grip strength is similar to previous studies (e.g., [27]). Binary logistic models for complex surveys were used to assess whether element blood concentrations were associated with the risk of low grip strength score (i.e., age- and sex-specific z-score below the 20th percentile). The same covariates were included as in GLMs (i.e., age, BMI, education, poverty to income ratio, alcohol, and smoking).

The sampling weights, strata, and primary sampling units created by the U.S. National Center for Health Statistics (NCHS) were applied to all statistical analyses according to NCHS guidelines to account for the complex, stratified multistage probability sample design of NHANES. All analyses were performed using R (version 3.5.0), using the packages survey and rms. *p*-values below 0.05 were considered to be statistically significant.

## 3. Results

In total, 6199 patients were included in our study: 3091 women (49.7%) and 3108 men (50.1%). Table 1 presents the characteristics of the study population by sex. The mean age was 45.9 (SD, 15.9) years for women and 45.8 (SD, 16.3) years for men. The average grip strength in men (mean, 88.9; SD, 17.8 kg) was much higher than that of women (57.0; 11.0 kg). Most participants reported never having smoked (66.5% of women and 49.1% of men). However, the majority of women and men reported drinking alcohol (58.8% and 80.4%, respectively).

Table 2 shows descriptive statistics on the concentrations of elements in blood of the women and men included in this study. Median concentrations were higher in men than women for lead, mercury, and selenium, but not for manganese. Blood lead median concentrations were 1.12 µg/dL and 1.68 µg/dL for women and men, respectively. Blood mercury median concentrations were 1.58 µg/L in women and 1.74 µg/L in men. The median blood selenium level was 193.4 µg/L in women and 198.2 µg/L in men. For manganese, women presented slightly higher blood levels than men, with median concentrations of 10.74 µg/L in women and 9.22 µg/L in men.

Table 3 presents the mean grip strength with respect to individual characteristics for men and women, respectively, and from univariate analysis. For both men and women, grip strength decreased significantly with age (*r* = −0.38 in men and −0.35 in women, indicating a decrease of 0.35 kg of grip strength per increase of 1 year of age in women for instance). When examining grip strength by age groups to allow for nonlinear relations, mean grip strength increased with age, peaking in the 30–39 years group, and then decreased in older age groups, similarly in men and women. Grip strength increased significantly with BMI in both sexes. Grip strength also varied significantly with education and race/ethnicity. Men and women in the lower education group (i.e., less than 9th grade) had poorer grip strength than those in the other groups. In addition, a stronger grip strength was observed in non-Hispanic black men and women compared with other race/ethnicity groups. In men, higher family income to poverty ratio was associated with stronger grip strength, but there was no such relation in women. Men and women who consumed alcohol had a lower grip strength than those who did not. Men and women who reported being regular smokers had stronger grip strength than the others.

The results of the GLMs for complex survey samples used to investigate associations between blood concentration of elements and grip strength are summarized in Table 4. A higher concentration of lead in blood was associated with significantly weaker grip strength in women (*p* < 0.05), with a 10-fold increase in lead being associated with lower grip strength by 2.37 kg (95% CI: −4.24, −0.50). Using the results showing that an increase in 1 year of age was associated with a 0.35 kg decrease in grip strength in women (Table 3), we could estimate that the −2.37 kg difference in grip strength is equivalent to about 6.8 years of aging (2.37/0.35). Blood lead was not associated with grip strength in men (β = 1.46 kg; 95% CI: −2.18, 5.10). Blood mercury concentration was not significantly associated with grip strength in either sex. The direction of the association estimate was positive for both men and women, indicating higher grip strength with higher blood mercury levels, but the confidence intervals were wide and far from significant. Higher blood selenium was significantly associated with stronger grip strength in women (β = 8.49 kg; 95% CI: 1.89, 15.10) and in men (β = 4.57 kg; 95% CI: −11.89, 21.03), but the association did not reach significance in the latter. Blood concentration of manganese was not significantly associated with grip strength in men or women.

**Figure 1:**
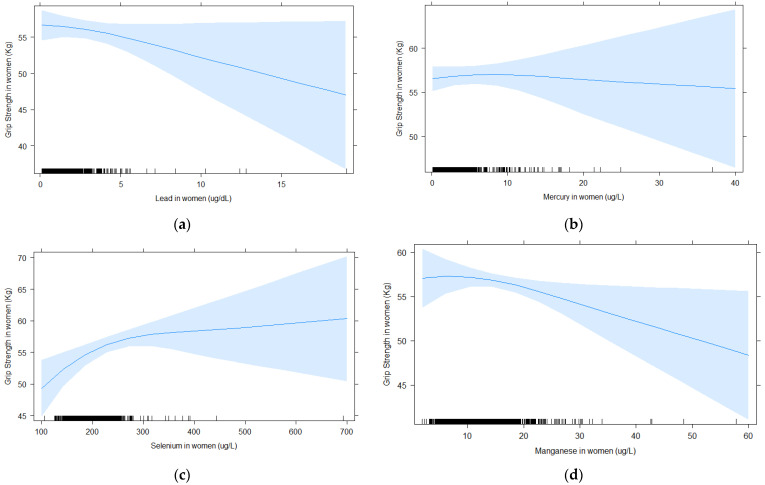
For women, associations between blood element concentrations and grip strength analyzed with splines (5 knots) for (**a**) lead, (**b**) mercury, (**c**) selenium, and (**d**) manganese. All models were adjusted for age, BMI, education, race/ethnicity, income to poverty ratio, smoking, and alcohol consumption.

**Figure 2:**
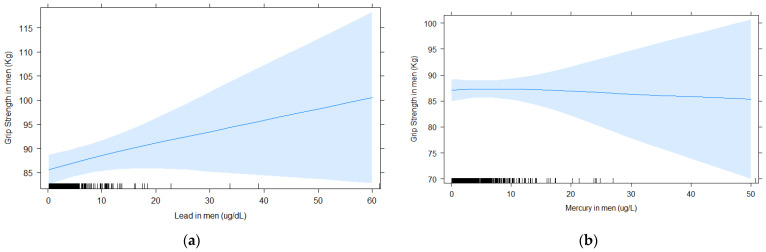

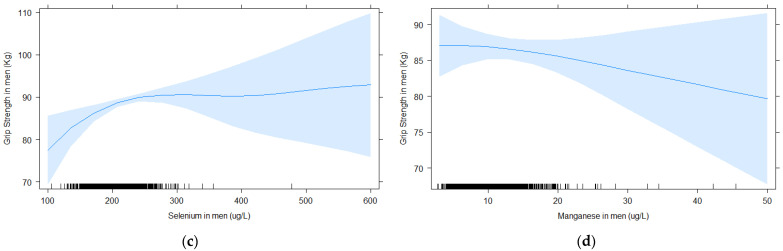
For men, associations between blood element concentrations and grip strength analyzed with splines (5 knots) for (**a**) lead, (**b**) mercury, (**c**) selenium, and (**d**) manganese. All models were adjusted for age, BMI, education, race/ethnicity, income to poverty ratio, smoking, and alcohol consumption.

Figure 1 and Figure 2 show the shape of the association between blood element concentrations and grip strength with cubic splines for women and men, respectively. It shows that the association between blood lead and grip strength in women appears linear, with a steady decrease in strength with increased blood lead levels (Figure 1a). For selenium in women, there is a steep increase in grip strength up until approximately 200 μg/L, then grip strength continues to increase with higher selenium, but the slope is less pronounced. The spline for blood mercury and grip strength was flat, indicating no association. In men, splines for blood lead and selenium suggest stronger grip strength with higher blood levels, whereas it was the reverse for manganese, but none of the association were very strong, consistent with the lack of significant associations in the GLM analysis. Similar to the results in women, the spline for blood mercury and grip strength was flat, indicating no association.

We also analyzed grip strength as a binary variable, categorizing participants as having ‘low’ or ‘normal’ grip strength (Table 5). Consistent with the GLM results, a 10-fold increase in blood lead was significantly associated with an elevated risk of having low grip strength among women (OR = 1.76; 95% CI: 1.09, 2.84). For selenium, a 10-fold increase in blood levels was significantly associated with a decreased risk of having a low grip strength among women (OR = 0.03; 95% CI: 0.003, 0.4). Blood concentrations of mercury and manganese showed no association in these analyses.

Finally, we ran GLMs including all four elements and the results were similar to those from the single-exposure models (Table 6). The association estimates between blood lead and weaker grip strength and that between blood selenium and stronger grip strength in women remained of similar magnitude and statistically significant. Likewise, manganese and mercury were not associated with grip strength in these models for mutually-adjusted elements.

## 4. Discussion

In the present study among adults from the general U.S. population, we observed that higher concentrations of lead were associated with weaker grip strength in women, and this association appeared to be approximately linear. We observed no association between blood lead and grip strength in men, similar to a previous study conducted on older men (i.e., >65 years) from the general population [4]. Other studies also assessed neuromotor function in relation to lead exposure, but relied on different tests for assessing this outcome. For instance, an analysis of older adults (i.e., >58 years, NHANES 1999–2002) reported that walking speed decreased significantly with increasing blood lead [6]. Similar to our study, this association was observed in women, whereas null findings were observed in men. The adverse effects of high occupational lead exposure on neuromotor function are well-documented [2,3,28], and the present study suggests that this might also apply to women from the general U.S. population. The magnitude of the association estimate for a ten-fold increase in blood lead levels was equivalent to about 6.8 years of aging. These results were obtained after controlling for several important potential confounders, including age and socioeconomic status.

With respect to mercury, we did not observe a significant association between blood levels of this metal and grip strength. Moreover, further exploration of the data for a potential threshold effect (e.g., an association appearing at the most extreme values of blood mercury concentrations) did not reveal any indication of an effect of mercury in this population. Previous studies linking adverse effects of mercury on neuromotor function among adults were conducted in populations with much higher levels of exposure owing to the frequent consumption of mercury-contaminated fish [29] or occupational exposure [30]. We did not find any other study investigating mercury exposure in relation to neuromotor function in the adult general population, hence there is no comparable investigation to place our findings into perspective. The lack of association between blood mercury and grip strength is likely due to the low levels of exposure experienced in this population and/or to the well-documented confusion bias introduced by the good nutrients present in fish such as omega-3 fatty acids and selenium. Fish and seafoods are the most important source of exposure to mercury in the U.S. population [31].

Our study suggests an association between low blood selenium levels and weaker grip strength in both men and women, although the results were significant only for the latter. Our results are consistent with those from previous studies showing associations between low blood selenium levels and weaker grip strength in older women living in Baltimore, Maryland [13] and in older adults living in Chianti, Italy [32]. The same results were observed in another study that evaluated other indicators of neuromotor function such as upper and lower limb coordination [12]. Furthermore, our findings showing that performance on motor function increases more steeply at lower than higher blood concentrations was also observed in another study conducted among Spanish and American adults [27]. It is noteworthy that all previous studies on selenium and neuromotor function were conducted on older populations, hence the present study extends these findings to individuals of younger age groups. The stronger hand grip observed in individuals with higher blood selenium could be due to the role of selenoproteins in muscular function. Skeletal muscles are important sites of selenium storage, and selenoproteins are known to be involved in muscle function. Hence, mutations in the gene causing deficiency in selenoprotein N are known to cause inherited neuromuscular disorders characterized by generalized muscle atrophy and muscle weakness [33]. Our findings showing that individuals with low blood selenium had weaker grip strength suggest that selenium deficiency might be prevalent in the general U.S. population, manifesting with poorer neuromotor function.

Like selenium, manganese is also an essential element for human health. However, we did not observe a significant association between blood manganese and grip strength in the present study. We have not been able to identify any other studies that have investigated grip strength in relation to blood manganese in the general population. However, populations with high environmental exposure to manganese because of their proximity to polluting industries might display neuromotor deficits [18,19,20]. Furthermore, our exploration of the shape of the exposure–response relation did not reveal other potential association, such as an inverse U-shaped curve, that may characterize essential nutrients. The lack of association between low blood manganese and grip strength is not surprising, as adverse health effects due to manganese deficiency in humans have only been reported under experimental conditions [34]. This element is essential, being a constituent of several metalloproteins [10], but dietary sources are common and provide sufficient intake for most, if not all individuals in the population.

An important strength of the present study is the large sample, being representativeness of the general, non-institutionalized population. In our analysis, we applied adequate methods for complex surveys to ensure that the findings are generalizable to the general U.S. population. All the analyses were adjusted for important confounders, and the results were robust when all four elements were included in the same model (i.e., results for mutually-adjusted elements). Further studies might be useful to explore whether interactions between these different elements might occur. Our study has some limitations, including the cross-sectional study design, which does not allow to assess the temporality of associations. Another limitation is our reliance on blood as a biomarker for manganese and mercury, as other biological matrices might be better biomarkers of exposure. For instance, a review and meta-analysis has concluded that hair was a better biomarker of exposure to manganese than blood [35]. Likewise, to the extent that the largest and most concerning form of mercury exposure is methylmercury, measurements made in hair might be more useful in future studies aimed at detecting neurofunctional effects resulting from overexposure to this metal [36]. Finally, the present study focused on hand grip strength because no other measure was available in the survey to assess neuromotor function. However, grip strength is a measure of particular interest because compelling evidence from longitudinal studies indicates that it is predictive of disability [37,38] and of mortality [39].

## 5. Conclusions

The findings from the present study indicate that higher blood lead was associated with weaker grip strength in women, even at the low exposure levels encountered in this population. Furthermore, low blood selenium levels were associated with weaker grip strength, suggesting that some individuals might have selenium deficiency manifesting with poorer neuromotor function. There was no indication that circulating concentrations of manganese and mercury were associated with neuromotor strength at the levels of exposure experienced by this population.

## Figures and Tables

**Table 1:** Description of the study population stratified by sex.

Characteristic	Women (*n* = 3091)	Men (*n* = 3108)
Age (years); mean (SD)	45.9 (15.9)	45.8 (16.3)
BMI (kg/m^2^); mean (SD)	29.3 (7.6)	28.4 (6.2)
Grip strength (kg); mean (SD)	57.0 (11)	88.9 (17.8)
Education level; *n* (%)		
Less than 9th grade	195 (6.3)	263 (8.5)
9–12th grade (no diploma)	374 (12.1)	424 (13.6)
High school graduate/GED equivalent	622 (20.1)	707 (22.8)
Some college or associate degree	1055 (34.1)	908 (29.2)
College graduate or above	845 (27.3)	806 (25.9)
Race/ethnic group; *n* (%)		
Non-Hispanic White	1192 (38.6)	1174 (37.8)
Non-Hispanic Black	744 (24.1)	758 (24.4)
Non-Hispanic Asian/other non-Hispanic	496 (16.1)	521 (16.8)
Mexican American and other Hispanic	659 (21.3)	655 (21.1)
Family income to poverty ratio; *n* (%)		
0.00–0.87	602 (19.5)	519 (16.7)
0.87–1.65	649 (20.9)	667 (21.5)
1.65–3.58	749 (24.2)	746 (24)
3.58–5.00	857 (27.7)	935 (30.1)
Missing data	234 (7.6)	241 (7.7)
Smoking status; *n* (%)		
Never	2057 (66.6)	1525 (49.1)
Occasional smoker	459 (14.9)	689 (22.2)
Regular smoker	493 (15.9)	806 (25.9)
Missing data	82 (2.6)	88 (2.8)
Alcohol consumption; *n* (%)		
No	1008 (32.6)	427 (13.7)
Yes	1816 (58.8)	2499 (80.4)
Missing data	267 (8.6)	182 (5.9)

**Table 2:** Descriptive statistics for element concentrations in blood for women (*n* = 3091) and men (*n* = 3108).

Blood Concentration	Min	Percentile 25	Median	Percentile 75	Max	SD	Geometric Mean
Lead (µg/dL)							
Women	0.11	0.55	0.88	1.36	19.4	1.06	0.89
Men	0.17	0.80	1.22	1.89	61.29	2.19	1.26
Mercury (µg/L)							
Women	0.11	0.42	0.82	1.76	36.99	2.29	0.89
Men	0.11	0.43	0.84	1.87	50.81	2.85	0.93
Selenium (µg/L)							
Women	105.4	177.2	191.0	206.3	734.8	28.88	191.6
Men	105.8	181.3	196.6	212.1	635.8	26.43	196.6
Manganese (µg/L)							
Women	1.88	7.82	9.85	12.69	62.51	4.40	10.02
Men	2.69	7.02	8.73	10.65	54.92	3.37	8.73

**Table 3:** Grip strength with respect to individual characteristics in women and men.

Characteristic	Women (*n* = 3091)	Men (*n* = 3108)
	Pearson’s r (IC95%)	
Age (years) ^1^	−0.35 (−0.38, −0.32)	−0.38 (−0.41, −0.35)
BMI (kg/m^2^) ^1^	0.20 (0.16, 0.23)	0.18 (0.15, 0.22)
	Mean (SD)	
Age groups (years) ^2^		
20–29	59.8 (10.3)	93.5 (17.3)
30–39	61.1 (10.4)	97.4 (17.4)
40–49	59.8 (11.1)	92.6 (15.6)
50–59	55.9 (9.8)	86.8 (15.3)
60–69	52.3 (9.1)	79.8 (16.1)
70–79	46.6 (9.5)	73.1 (13.4)
Education level ^2^		
Less than 9th grade	50.8 (10.4)	78.9 (16.5)
9–12th grade (no diploma)	55.5 (11.1)	88.0 (19.3)
High school graduate/GED equivalent	56.4 (11.3)	90.4 (17.1)
Some college or associate degree	58.2 (11.2)	91.1 (17.7)
College graduate or above	58.1 (10.2)	88.9 (17.1)
Race/ethnicity ^2^		
Non-Hispanic White	57.1 (10.7)	90.8 (17.5)
Non-Hispanic Black	61.9 (11.6)	93.2 (18.7)
Non-Hispanic Asian/other non-Hispanic	53.6 (9.8)	83.5 (16.1)
Mexican American and other Hispanic	53.9 (9.8)	84.8 (16.6)
Family income to poverty ratio ^3^		
0.00–0.87	57.3 (11.7)	86.7 (18.1)
0.87–1.65	56.5 (11.5)	88.8 (18.4)
1.65–3.58	57.2 (11.1)	89.6 (18.1)
3.58–5.00	57.8 (10.3)	90.7 (16.8)
Missing	54.5 (10.0)	84.6 (17.9)
Smoking status ^2^		
Never	56.6 (11.0)	89.9 (17.9)
Occasional smoker	56.6 (10.8)	84.8 (16.8)
Regular smoker	58.7 (11.3)	91.2 (17.7)
Missing	60.3 (10.8)	84.6 (17.9)
Alcohol consumption ^2^		
No	58.1 (10.8)	89.9 (17.5)
Yes	55.1 (11.4)	84.8 (18.5)
Missing	60.3 (10.8)	83.0 (19.9)
All participants	57.0 (11.1)	88.9 (17.8)

^1^ Significant association between age and BMI and grip strength for both sexes (*p* < 0.001). ^2^ Significant difference between characteristic’s groups and grip strength for both sexes (*p* < 0.001). ^3^ In women, there was no significant difference in mean grip strength with respect to family income to poverty ratio (*p* = 0.28), but the difference was significant in men (*p* < 0.001).

**Table 4:** Change in grip strength for a 10-fold increase in the concentration of each blood element in men and women.

Blood Concentration	Women (*n* = 2609)		Men (*n* = 2706)	
	β (CI95%)	*p*-Value	β (CI95%)	*p*-Value
Lead (µg/dL)	−2.37 (−4.24, −0.50)	0.03	1.87 (−1.69, 5.43)	0.32
Mercury (µg/L)	0.18 (−0.89, 1.26)	0.75	1.38 (−0.22, 2.98)	0.11
Selenium (µg/L)	8.49 (1.89, 15.10)	0.02	4.57 (−11.89, 21.03)	0.59
Manganese (µg/L)	−2.08 (−4.76, 059)	0.15	−1.49 (−5.87, 2.88)	0.51

Note: Estimates are from GLMs for complex survey design for the association between blood concentrations of elements separately and grip strength, adjusting for age, BMI, education, race/ethnicity, income to poverty ratio, smoking, and alcohol consumption.

**Table 5:** Odds of low grip strength for a 10-fold increase in blood concentration of elements.

Blood Concentration	Women (*n* = 2609)	Men (*n* = 2706)
	OR (95% CI)	OR (95% CI)
Lead (µg/dL)	1.76 (1.09, 2.84)	0.87 (0.58, 1.30)
Mercury (µg/L)	0.96 (0.69, 1.32)	0.77 (0.55, 1.06)
Selenium (µg/L)	0.03 (0.003, 0.4)	3.39 (0.27, 41.38)
Manganese (µg/L)	0.83 (0.38, 1.78)	1.53 (0.64, 3.67)

Note: Results are from logistic regressions for complex survey design for the association between blood concentration of elements and low grip strength (i.e., grip strength ≤ 20th percentile), adjusting for age, BMI, education, race/ethnicity, income to poverty ratio, smoking, and alcohol consumption.

**Table 6:** Change in grip strength for a 10-fold increase in the concentration of blood elements, from models including all four elements.

Blood Concentration	Women (*n* = 2609)		Men (*n* = 2706)	
	β (CI95%)	*p*-Value	β (CI95%)	*p*-Value
Lead (µg/dL)	−2.50 (−4.40, −0.61)	0.02	1.46 (−2.18, 5.10)	0.45
Mercury (µg/L)	0.34 (−0.72, 1.39)	0.54	1.31 (−0.36, 2.97)	0.15
Selenium (µg/L)	8.19 (1.69, 14.69)	0.03	3.67 (−12.79, 20.14)	0.67
Manganese (µg/L)	−1.84 (−4.48, 0.79)	0.20	−1.87 (−6.37, 2.62)	0.43

Estimates are from GLMs for complex survey design for the association between blood concentrations of elements and grip strength, adjusting for age, BMI, education, race/ethnicity, income to poverty ratio, smoking, and alcohol consumption.

## Data Availability

The data used in the present study are available at https://wwwn.cdc.gov/nchs/nhanes/Default.aspx (accessed on 3 November 2018).
